# Occurrence of genes of putative fibrinogen binding proteins and hemolysins, as well as of their phenotypic correlates in isolates of *S. lugdunensis *of different origins

**DOI:** 10.1186/1756-0500-4-113

**Published:** 2011-04-08

**Authors:** Florian Szabados, Yasmina Nowotny, Lennart Marlinghaus, Miriam Korte, Sandra Neumann, Martin Kaase, Sören G Gatermann

**Affiliations:** 1 Institute for Hygiene and Microbiology, Dept. for Medical Microbiology, University Bochum Universitätsstraße 150, Bochum, Germany

## Abstract

**Background:**

*Staphylococcus lugdunensis *is an important human pathogen that causes potentially fatal endocarditis, osteomyelitis and skin and soft tissue infections similar to diseases caused by *Staphylococcus aureus*. Nevertheless, in contrast to *S. aureus*, data on pathogenicity factors of *S. lugdunensis *is scarce. Two adhesins, a fibrinogen and a von Willebrand factor binding protein, and a *S. lugdunensis *synergistic hemolysin (SLUSH) have been previously described. Moreover, the newly sequenced genome of *S. lugdunensis *revealed genes of other putative fibrinogen binding adhesins and hemolysins. The aim of this study was to gain more insight into the occurrence of genes likely coding for fibrinogen binding adhesins and hemolysins using clinical strains of *S. lugdunensis*.

**Findings:**

Most of the putative adhesin genes and hemolysin genes investigated in this study were highly prevalent, except for the SLUSH gene cluster. In contrast to previous reports, binding to fibrinogen was detected in 29.3% of the *S. lugdunensis *strains. In most strains, hemolysis on blood agar plates was weak after 24 h and distinct after 48 h of incubation. The fibrinogen binding and hemolysis phenotypes were also independent of the type of clinical specimen, from which the isolates were obtained.

**Conclusion:**

In this study we described a pyrrolidonyl arylamidase negative *S. lugdunensis *isolate. Our data indicate that a matrix-assisted laser desorption ionisation time-of-flight MS-based identification of *S. lugdunensis *or species-specific PCR's should be performed in favour of pyrrolidonyl arylamidase testing. In contrast to the high occurrence of putative fibrinogen binding protein genes, 29.3% of the *S. lugdunensis *strains bound to fibrinogen. Putative hemolysin genes were also prevalent in most of the *S. lugdunensis *strains, irrespective of their hemolysis activity on Columbia blood agar plates. Similar to a previous report, hemolysis after 48 h of incubation is also indicative for *S. lugdunensis*. The SLUSH gene cluster was detected in an estimated 50% of the strains, indicating that this locus is different or non-prevalent in many strains.

## Background

*Staphylococcus lugdunensis *is an important human pathogen that causes potentially fatal endocarditis, osteomyelitis and skin and soft tissue infections (SSTI) similar to diseases caused by *S. aureus *[1-5]. Fibrinogen and fibronectin binding adhesins have been discussed as a pathogenicity factor of *S. aureus *[6,7]. In *S. lugdunensis*, two adhesins, the fibrinogen binding protein (Fbl) [8-10] and the von Willebrand factor binding protein [11] have been described. A hemolysin, the *S. lugdunensis *synergistic hemolysin (SLUSH), has also been described [12,13]. The newly sequenced genome [14] of *S. lugdunensis *has revealed an additional gene (SLGD_01696) that has been annotated as a putative fibrinogen/fibronectin binding adhesin [15]. Nevertheless, data on the prevalence of *S. lugdunensis *adhesins and hemolysins, in contrast to *S. aureus*, is scarce. We therefore designed primers (Table 1) to characterize the occurrence of genes coding for putative fibrinogen binding proteins and supposed hemolysins.

**Table 1:** Primers used for detection

Gene/locus-tag	Name	Sequence 5' 3'	Size (bp)	Primer
*fbl*	fbl_check_F	CGTATTATCCCAAGTAGCAACC	404	This study
	fbl_check_R	CTTCATCGATTGTCCCAGTAGC		

SLGD_01696	FbpA_F	GAGATTACTGGACAACAAACG	558	This study
	FbpA_R	GTATTGTGACGTCGTTTCCTG		

SLGD_00006	betahemolysin_F	TGGTCAAGGTACAGAAGGTTGGCA	449	This study
	betahemolysin_R	TATCCCAACTATACGCGTTGCCCT		

SLGD_00847	hemolysinIII_F	TAATGCTGTTTCGCACGGAGTTGC	407	This study
	hemolysinIII_R	GACGCCTACCCATCCCATTACAA		

SLUSH-cluster	slush_donvito_F	TTTCGTCTTTGCACACACATTTCCA	977	This study
	slush_donvito_R	ACAGCACAAAGCCTTAACTATCTCA		

SLGD_02429	stlu_vwbl_F	TGGCGGGATGATTTGGACGGG	858	This study
*vwbl*	stlu_vwbl_R	TCGCCTTCTTGCCCTGATGGT		

The previously published fibrinogen binding protein gene (*fbl*) sequences [8,9], the von Willebrand factor binding protein precursor gene (*vwbl*) sequences (AY530288) [18] and SLGD_02429 [13], the putative fibrinogen/fibronectin binding protein (FbpA homologue SLGD_01696) gene sequence [13], the *S. lugdunensis *synergistic hemolysin (SLUSH) gene sequence (U73444.1) [11], the *S. lugdunensis *putative beta-hemolysin (SLGD_00006) gene sequence [11] and the *S. lugdunensis *putative hemolysin III (SLGD_00847) gene sequence [11] were used to design primer pairs (This study).

## Methods

### Bacteria

Fifty-eight clinical strains of *S. lugdunensis *representing single patient isolates collected non-consecutively between 2003 and 2008 were included in this study (Table 2). This collection represents both urban and rural settings from the Bochum area, as well as a variety of community and institutional facilities. *S. lugdunenis *was preliminary identified by typical characteristics, such as odor, and the GPI-card by the Vitek-2 automated identification system (bioMérieux, Marcy l'Etoile, France). In addition, the strains were tested for the presence of ornithine decarboxylase (ODC), an enzyme that catalyzes the decarboxylation of ornithine to form putrescine. Retrospectively, the presence of the pyrrolidonyl arylamidase (PYR), which hydrolyzes L-pyrrolidonyl-ß-naphtylamide to L-pyrrolidone and ß-naphtylamide, was also tested. The *S. lugdunensis *type strain DSM 4804 was used as a positive control in both tests. The species diagnosis was confirmed using matrix-assisted laser desorption/ionization time-of-flight (MALDI-TOF) mass spectrometry (MS) [16] and amplification of the *tanA *gene, as previously described [17]. A single isolate was also confirmed by sequencing of the *sodA *gene, as previously described [18] Three strains of *S. epidermidis *(n = 2) and *S. hominis *(n = 1) initially misidentified as *S. lugdunensis *were excluded from this study.

**Table 2:** Occurrence of putative fibrinogen binding protein and hemolysin genes and their phenotypic correlates

Strains	Type of culture	Species specific *tanA *gene	PYR	ODC	Clumping factor	Binding to solid-phase fibrinogen	Slidex Staph plus	*fbl*	SLGD_01696 FbpA	*vwbl*	Hemolysis on Columbia blood agar after 24 h	Hemolysis on Columbia blood agar after 48 h	SLGD_00847 hemolysin III	SLGD_00006 beta-hemolysin	SLUSH gene cluster
Stlu 12	blood culture	**+**	**+**	**+**	**+**	**+**	**+**	**+**	**+**	-	-	**+**	**+**	**+**	-

Stlu 17	blood culture	**+**	**+**	**+**	-	-	-	**+**	**+**	**+**	-	**+**	**+**	**+**	-

Stlu 20	blood culture	**+**	**+**	**+**	-	-	(+)	**+**	**+**	**+**	**+**	**++**	**+**	**+**	-

Stlu 21	blood culture	**+**	**+**	**+**	**+**	**+++**	**+**	**+**	**+**	**+**	-	**+**	**+**	**+**	**+**

Stlu 22	blood culture	**+**	**+**	**+**	-	-	-	**+**	**+**	**+**	-	**+**	**+**	**+**	**+**

Stlu 25	blood culture	**+**	**+**	**+**	-	-	-	**+**	**+**	**+**	-	**+**	**+**	**+**	**+**

Stlu 27	blood culture	**+**	**+**	**+**	**+**	**+**	**+**	**+**	**+**	**+**	-	**+**	**+**	**+**	**+**

Stlu 28	blood culture	**+**	**+**	**+**	-	-	-	**+**	**+**	**+**	-	-	**+**	**+**	**+**

Stlu 30	blood culture	**+**	**+**	**+**	-	-	-	**+**	**+**	**+**	-	**+**	**+**	**+**	**+**

Stlu 32	blood culture	**+**	**+**	**+**	**+**	**+**	**+**	**+**	**+**	**+**	-	**+**	**+**	**+**	**+**

Stlu 33	blood culture	**+**	**+**	**+**	-	-	-	**+**	**+**	**+**	-	**+**	**+**	**+**	**+**

Stlu 34	blood culture	**+**	±*	**+**	-	-	-	**+**	**+**	**+**	-	**+**	**+**	**+**	**+**

Stlu 39	blood culture	**+**	**+**	**+**	-	-	-	**+**	**+**	**+**	-	**+**	**+**	**+**	**+**

Stlu 50	blood culture	**+**	**+**	**+**	**+**	**++**	**+**	**+**	**+**	**+**	-	**+**	**+**	**+**	-

Stlu 53	blood culture	**+**	**+**	**+**	**+**	**+**	**+**	**+**	**+**	**+**	-	**+**	**+**	**+**	-

Stlu 58	blood culture	**+**	**+**	**+**	**+**	**+**	**+**	**+**	**+**	**+**	**+**	**+**	**+**	**+**	-

															

Stlu 2	wound swab	**+**	**+**	**+**	-	-	-	**+**	**+**	**+**	-	**+**	**+**	**+**	**+**

Stlu 3	wound swab	**+**	**+**	**+**	-	-	-	**+**	**+**	**+**	-	**+**	**+**	**+**	-

Stlu 5	wound swab	**+**	**+**	**+**	-	-	-	**+**	**+**	**+**	-	**+**	**+**	**+**	**+**

Stlu 6	wound swab	**+**	**+**	**+**	**+**	**+++**	**+**	**+**	**+**	**+**	-	**+**	**+**	**+**	-

Stlu 7	wound swab	**+**	-	**+**	**+**	**+**	**+**	**+**	**+**	**+**	-	**+**	**+**	**+**	-

Stlu 8	wound swab	**+**	**+**	**+**	**+**	**+++**	**+**	**+**	**+**	**+**	-	**+**	**+**	-	-

Stlu 10	wound swab	**+**	**+**	**+**	-	-	-	**+**	**+**	**+**	-	**+**	**+**	**+**	-

Stlu 11	wound swab	**+**	**+**	**+**	-	-	-	**+**	**+**	**+**	-	**+**	**+**	**+**	-

Stlu 13	wound swab	**+**	**+**	**+**	**+**	**+**	(+)	**+**	**+**	**+**	-	**+**	**+**	**+**	**+**

Stlu 14	wound swab	**+**	**+**	**+**	-	-	-	**+**	**+**	**+**	-	**+**	**+**	**+**	-

Stlu 15	wound swab	**+**	**+**	**+**	**+**	**+**	**+**	**+**	**+**	**+**	-	**+**	**+**	**+**	-

Stlu 16	wound swab	**+**	**+**	**+**	-	-	-	**+**	**+**	**+**	-	**+**	**+**	**+**	-

Stlu 23	wound swab	**+**	**+**	**+**	-	-	-	**+**	**+**	**+**	-	**+**	**+**	**+**	**+**

Stlu 24	wound swab	**+**	**+**	**+**	-	-	-	**+**	**+**	**+**	-	**+**	**+**	**+**	**+**

Stlu 26	wound swab	**+**	**+**	**+**	-	-	-	**+**	**+**	**+**	-	-	**+**	**+**	**+**

Stlu 29	wound swab	**+**	**+**	**+**	-	-	-	**+**	**+**	**+**	-	**+**	**+**	**+**	**+**

Stlu 35	wound swab	**+**	**+**	**+**	-	-	-	**+**	**+**	**+**	-	**+**	**+**	**+**	**+**

Stlu 36	wound swab	**+**	**+**	**+**	-	-	-	**+**	**+**	**+**	-	**+**	**+**	**+**	**+**

Stlu 37	wound swab	**+**	**+**	**+**	-	-	-	**+**	**+**	**+**	**+**	**+**	**+**	**+**	-

Stlu 38	wound swab	**+**	**+**	**+**	-	-	-	**+**	**+**	**+**	-	**+**	**+**	-	**+**

Stlu 40	wound swab	**+**	**+**	**+**	-	-	-	**+**	**+**	**+**	-	**+**	**+**	**+**	-

Stlu 41	wound swab	**+**	**+**	**+**	**+**	**+++**	**+**	**+**	**+**	**+**	-	**+**	**+**	**+**	-

Stlu 42	wound swab	**+**	**+**	**+**	**+**	**+**	**+**	**+**	**+**	**+**	-	**+**	**+**	**+**	**+**

Stlu 43	wound swab	**+**	**+**	**+**	-	-	-	**+**	**+**	**+**	-	**+**	**+**	**+**	**+**

Stlu 44	wound swab	**+**	**+**	**+**	-	-	-	**+**	**+**	**+**	-	**+**	**+**	**+**	-

Stlu 45	wound swab	**+**	**+**	**+**	**+**	**++**	**+**	**+**	**+**	**+**	-	**+**	**+**	**+**	**+**

Stlu 49	wound swab	**+**	**+**	**+**	-	-	-	**+**	**+**	**+**	-	**+**	**+**	**+**	-

Stlu 52	wound swab	**+**	**+**	**+**	**+**	**+**	(+)	**+**	**+**	**+**	-	**+**	**+**	**+**	-

Stlu 54	wound swab	**+**	**+**	**+**	-	-	-	**+**	**+**	**+**	-	**+**	**+**	**+**	-

Stlu 55	wound swab	**+**	**+**	**+**	-	-	-	**+**	**+**	**+**	**+**	**++**	**+**	**+**	-

Stlu 57	wound swab	**+**	**+**	**+**	-	-	-	**+**	**+**	**+**	-	**+**	**+**	**+**	-

Stlu 59	wound swab	**+**	**+**	**+**	-	-	-	**+**	**+**	**+**	-	**+**	**+**	**+**	-

Stlu 60	wound swab	**+**	**+**	**+**	-	-	-	**+**	**+**	**+**	-	**+**	**+**	**+**	**+**

Stlu 61	wound swab	**+**	**+**	**+**	-	-	-	**+**	**+**	**+**	-	-	**+**	**+**	**+**

Stlu 9	cathether	**+**	**+**	**+**	-	-	-	**+**	**+**	**+**	-	**+**	**+**	**+**	-

Stlu 19	cathether	**+**	**+**	**+**	-	-	-	**+**	**+**	**+**	**+**	**++**	**+**	**+**	**+**

Stlu 1	knee aspirate	**+**	**+**	**+**	**+**	**+**	-	**+**	**+**	**+**	-	**+**	**+**	**+**	**+**

Stlu 4	knee aspirate	**+**	**+**	**+**	-	-	-	**+**	**+**	**+**	-	**+**	**+**	**+**	-

Stlu 18	knee aspirate	**+**	**+**	**+**	-	-	-	**+**	**+**	**+**	-	**+**	**+**	**+**	-

Stlu 31	knee aspirate	**+**	±*	**+**	-	-	-	**+**	**+**	**+**	-	**+**	**+**	**+**	**+**

Stlu 51	knee aspirate	**+**	**+**	**+**	-	-	-	**+**	**+**	**+**	-	**++**	**+**	**+**	-

Stlu 48	bone aspirate	**+**	**+**	**+**	-	-	-	**+**	**+**	**+**	-	**+**	**+**	**+**	-

Fifty-eight clinical strains of *S. lugdunensis *representing single patient isolates were used in this study. The strains were characterized with regard to putative fibrinogen binding and hemolysin genes and compared to their phenotypic correlates. Strains with a complete hemolysis were interpreted as positive (+). Strains producing no hemolysis (-) were compared to the negative control *S. carnosus *TM 300. Strains with a strong hemolysis after 48 h similar to that of *S. aureus *Cowan I were interpreted as producing strong hemolysis (++); + = positive; - = negative; (+) = weakly positive; ++ = strongly positive; ±* = test result indeterminate; pyrrolidonyl arylamidase (PYR); ornithine decarboxylase (ODC).

### PCR

Strains were molecularly characterized with regard to genes coding for putative fibrinogen binding proteins, such as *fbl*, SLGD 1696 (FbpA), a von Willebrand factor binding protein gene *vwbl*, and genes coding for putative hemolysins [14]. The published sequences were used [8,9] to design a primer pair for the detection of the *fbl *gene (Table 1). The von Willebrand factor binding protein precursor gene (*vwbl*) sequences (AY530288) [11] and SLGD_02429 [14] were used to design a primer pair for the detection of the *vwbl *gene. The annotated putative fibrinogen/fibronectin binding protein (SLGD_01696) gene sequence [14] was used to design primers to amplify this gene (Table 1). The SLUSH gene sequence (U73444.1) [12], the annotated putative beta-hemolysin (SLGD_00006) gene sequence [14] and the annotated putative hemolysin III (SLGD_00847) gene sequence [14] were used to design primers for the detection of the beta-hemolysin and hemolysin III genes (Table 1). Genomic DNA of *S. lugdunensis *was extracted using the QIAamp DNA Mini Kit (Quiagen, Hilden, Germany) suspending the bacteria in the recommended buffer and addition of 20 μg lysostaphin at the lysis step. The master mix contains dNTP's (dATP, dCTP, dGTP, and dTTP), 2 units of pure Taq DNA polymerase and reaction buffer. The reaction contained also 12.5 pmoles of each primer, 5 μl of 1:10 diluted template DNA, and water to 25 μl. All PCRs used an initial denaturation at 94°C for 5 min, followed by 35 cycles of an annealing step at 55°C for 30 s, an elongation step at 72°C for 60 s and denaturation step at 94°C for 30 s.

### Bacterial adherence to solid-phase fibrinogen

Binding of bacterial cells to immobilized fibrinogen was previously described [19]. Briefly, the strains were grown in 4 ml LB-medium for 18 h at 37°C and 100 rpm. A 96-well microtiter plate was coated with 20 μg/ml of purified fibrinogen (plasminogen, von Willebrand factor and fibronectin depleted, Enzyme Research Laboratories, South Bend, USA) in PBS (NaCl 137 mM, KCl 2.7 mM, Na_2_HPO_4_ 10 mM, KH_2_PO_4_ 1.76 mM, pH 7.4) for 18 h at 4°C. After the solution had been discarded, bovine serum albumin (2%, w/v, in PBS) was added for 1 h at 37°C. The plates were washed three times with PBS; then 100 μl of a cell suspension (washed two times in PBS) of OD600 = 1.0 was added and incubated at room temperature for 2 h. The wells were washed three times with PBS and bound cells were fixed with 100 μl formaldehyde (25%, v/v) for 30 min at room temperature. Adherent cells were washed with PBS twice, stained with crystal violet (0.5%, v/v) for 10 min at room temperature, then washed with PBS again, and dried at 37°C. Absorbance was measured at 550 nm in an ELISA plate reader (Bio Kinetics Reader EL340, Bio-Tek Instruments). An OD_550 _value of 0 to 0.06 was interpreted as negative, 0.07 to 0.15 as intermediately positive (+), 0.15 to 0.3 as positive (++), and >0.3 as strongly positive (+++). The *S. aureus *Newman (ATCC 25904) was used as a positive control and tested strongly positive (+++). A sample without bacteria was used to determine the background of this assay.

### Clumping factor and latex agglutination

All strains of the *S. lugdunensis *collection were tested for agglutination using the commercial *S. aureus *agglutination kit Slidex Staph Plus (bioMérieux). For detection of fibrinogen clumping a single colony of *S. lugdunensis *was stirred into 50 μl of human citrate plasma and then placed onto a glass slide. An agglutination within 60 s was interpreted as a positive result (Table 2). The *S. aureus *Newman (ATCC 25904) was used as a positive control.

### Hemolysis on Columbia blood agar plates

Bacteria were plated from the frozen stock cultures onto Columbia blood agar plates with 5% sheep blood (bioMérieux) and were grown for 24 h and 48 h of incubation at 37°C. In order to characterize the grade of the beta-hemolysis induced by *S. lugdunensis*, the distinct hemolysis was compared to that of an *S. carnosus *TM 300 and *S. aureus *Cowan I. Strains with a complete hemolysis after 24 h of incubation were interpreted as positive compared to the negative control *S. carnosus *TM 300 (ATCC 51365). Strains with a hemolysis after 48 h of incubation similar to that of *S. aureus *Cowan I (ATCC 12598) were interpreted as producing strong hemolysis.

## Results and Discussion

For *S. aureus *identification, agglutination-based kits are often used. These kits usually include antibodies against fibrinogen binding proteins. It has been described that some strains of *S. lugdunensis *react in these agglutination tests [20]. The correct identification of *S. lugdunensis *is thus difficult. For the identification of *S. lugdunensis *the determination of PYR and ODC has been described to be sufficient to distinguish *S. lugdunensis *from other Staphylococci [1,20]. Moreover, a new species, *Staphylococcus pseudolugdunensis*, has been recently described, which was also PYR and ODC positive [21]. In addition, PYR negative isolates of *S. lugdunensis *exist. Isolate Stlu 7 (Table 2) was PYR negative and ODC positive. The identification was confirmed by MALDI-TOF MS [15], by sequencing of the *sodA *gene [17], and also by the detection of the species specific *fbl *and *tanA *genes as previously described. In two other isolates the PYR test was indeterminate (Table 2). Since only 94.8% of *S. lugdunensis *isolates were PYR positive, an identification strategy based solely on the determination of the PYR activity could miss an estimated 5% of the *S. lugdunensis *isolates. In addition, the fibrinogen binding gene *fbl *has been used to establish a *S. lugdunensis *species-specific PCR [22,23]. In our strain collection all 58 strains were shown to be *fbl *positive using a primer set targeting the A-region of the *fbl *gene. This prevalence is similar to the high occurrence of the *fbl *previously described [22,23]. All *S. lugdunensis *strains were *tanA *positive (100%) and all were identified as *S. lugdunensis *by MALDI-TOF MS (100%). Our data suggest using MALDI-TOF MS technology or detection of the *fbl *or *tanA *gene for the identification of *S. lugdunensis *and argues against PYR testing for *S. lugdunensis *identification.

### Fibrinogen binding

Fibrinogen binding ability is usually targeted in *S. aureus *identification assays such as the Slidex Staph plus (bioMérieux) and the clumping factor test. It has been previously described that some strains of *S. lugdunensis *could be misidentified as *S. aureus *using these tests [20]. In our strains, 17 out of 58 (29.3%) were positive in the clumping factor test and 17 out of 58 (29.3%) strains were also positive in the *S. aureus *identification kit Slidex Staph plus. Expectedly, all of the strains that reacted in the clumping factor bound to solid phase fibrinogen, too. One isolate (Stlu 20) reacted in the Slidex Staph plus test but did not bind to soluble and solid phase fibrinogen. In another isolate (Stlu 1), binding to soluble and solid phase fibrinogen was detected but no agglutination was observed in the Slidex Staph plus test (Table 2). It has been recently reported that *S. aureus *virulence in sepsis is facilitated by the multiple repeats within an fibrinogen and fibronectin binding adhesin [24]. In the subgroup of blood culture samples 43.7% of the strains bound to fibrinogen compared to 26.5% in the subgroup of wound swabs. Nevertheless, the difference was statistically not significant as tested by Fischer's exact test. Differences in fibrinogen binding may be explained by different expression of the fibrinogen binding proteins. Moreover, the production of an extra cellular matrix, such as a capsule, which has been described for *S. lugdunensis *[2] could also mask the accession of an adhesin. Similar results has been described for *S. aureus*: fibrinogen binding greatly varies within the strains [25]. In contrast to previous reports, where 64.7% [26] and 83.7% [27] had been described as clumping factor positive 29.3% of our strains would be misidentified as *S. aureus *in the clumping factor and in the Slidex Staph plus agglutination test. In all strains, the genes for the FbpA-homologue (SLGD_01696) and *fbl *were present and, therefore we cannot judge if FbpA is involved in fibrinogen binding. Whether *fbl *or other fibrinogen binding proteins react in these assays warrants confirmation in future studies. The occurrence of the *vwbl *gene was also high (98.2%), similar to a previous report [18].

No statistical difference was observed in our study with regard to the type of clinical specimen from which the isolate was obtained and the fibrinogen binding phenotype. A cluster analysis was performed, but also did not show any correlation of bacterial phenotype and sample source (data not shown).

### Beta-hemolysis

The ability to induce beta-hemolysis on Columbia blood agar plates (bioMérieux) after 24 h incubation and 48 h incubation was investigated. The beta-hemolysis of most strains of *S. lugdunensis *was weaker compared to *S. aureus*. Only a hemolysin similar to delta hemolysin of *S. aureus *has been described for *S. lugdunensis *[28]. In addition, synergistic hemolysins have also been described [11,12,29]. Similar to findings by others, virtually all strains showed hemolysis after 48 h of incubation [30]. The hemolysis of red blood cells is an important pathogenicity factor. Expectedly, the iron level is an important regulator for hemolysin expression in many bacteria such as *Serratia marcescence *[31] and *E. coli *[32]. Fifty-four of 58 strains (93.1%) showed no hemolysis after 24 h. After 48 h three strains also showed no hemolysis, two strains showed a weak hemolysis, 49 showed distinct hemolysis and four showed a strong hemolysis, similar to the hemolysis induced by *S. aureus *after 48 h. In all strains the gene for hemolysin III was found. Fifty-six out of 58 strains were also positive for the beta-hemolysin gene. Interestingly, 30 out of 58 strains (only 51.7%) were negative for the PCR detecting the SLUSH gene cluster. The initially described [11] SLUSH gene cluster (SLUSH A to C) was annotated as methylated-DNA protein-cysteine methyltransferase (SLGD_00440), and has an identity of 89% in the newly sequenced *S. lugdunensis *genome [13]. The gene SLGD_00441 was annotated as SLUSH B with an identity of 92% to the previously described gene cluster [11]. The following gene SLGD_00442 was also annotated as "SLUSH B" with an identity of 87% to the previously described gene cluster [11]. Interestingly, three out of four strains that were strongly positive with regard to phenotypic hemolysis were negative in the SLUSH gene specific PCR (Table 2). Notably, SLUSH gene-derived proteins have also been described as phenol soluble modulins [33]. This indicates that the SLUSH gene locus could be different or non-prevalent in an approximately 50% of the strains and therefore the SLUSH gene cluster and the basis of hemolysis should be characterized in further detail. No statistical difference was observed in our study with regard to the type of clinical specimen from which the isolate was obtained and the hemolysis phenotype.

## Conclusion

In this study we described a PYR negative isolate of *S. lugdunensis*. Our data indicate that a matrix-assisted laser desorption ionisation time-of-flight MS-based identification of *S. lugdunensis *or species-specific PCR's, detecting the *fbl *or the *tanA *gene, should be used in favour of a PYR testing. In addition, this study provides a first look into the occurrence of putative fibrinogen-binding proteins and suspected hemolysins of *S. lugdunensis *and their phenotypic correlates. The *fbl*, the FbpA-homologue (SLGD 1696) and the *vwbl *are highly prevalent in *S. lugdunensis *strains. Notably, this high occurrence was independent of the fibrinogen binding ability of the strains. In contrast to previous reports only 29.3% of the strains bound to fibrinogen. Such strains could be misidentified as *S. aureus *in fibrinogen binding-based agglutination tests. Genes coding for hemolysins (hemolysin III [SLGD_00847] and beta-hemolysin [SLGD_00006]) are also highly prevalent in the strains except for the SLUSH gene cluster, which was detected in only an approximately 50% of the strains. This indicates that the SLUSH gene locus could be different or non-prevalent among many strains. The fibrinogen binding and hemolysis phenotypes were independent of the type of clinical specimen from which the isolates were obtained.

## Competing interests

The authors declare that they have no competing interests.

## Authors' contributions

FS conceived and coordinated this study in addition to drafting the manuscript. YN, LM, SN and MiK carried out the molecular characterization and agglutination tests. FS, MaK and SG edited the manuscript. All authors read and approved the final manuscript.
